# *TP53*-mutated MDS and AML: immune dysregulation, tumor microenvironment, and emerging therapeutic strategies

**DOI:** 10.3389/fonc.2025.1655486

**Published:** 2025-08-20

**Authors:** Marwah M. Albakri

**Affiliations:** ^1^ Department of Clinical Laboratory Sciences, College of Applied Medical Sciences, Taibah University, Madinah, Saudi Arabia; ^2^ Health and Life Research Center, Taibah University, Madinah, Saudi Arabia

**Keywords:** TP53 mutation, myelodysplastic syndromes (MDS), acute myeloid leukemia (AML), immune evasion, tumor microenvironment, targeted therapies

## Abstract

*TP53* mutations drive oncogenesis and therapeutic resistance in myelodysplastic syndromes (MDSs) and acute myeloid leukemia (AML), impairing p53-regulated functions such as apoptosis, immune surveillance, and genomic stability, leading to immune evasion and metabolic reprogramming. The tumor microenvironment in *TP53*-mutated MDS and AML fosters leukemic progression through cytokine dysregulation, altered metabolism, and immune suppression. Current therapies, including chemotherapy and hypomethylating agents, offer limited efficacy, resulting in poor overall survival rates for these high-risk patients. However, novel therapeutic approaches provide promising avenues, including MDM2 inhibitors, p53-reactivating agents, pathway-targeted inhibitors (Hedgehog, Wnt, NF-κB), immune modulation (checkpoint inhibitors, CAR-T therapy), metabolic interventions (fatty acid metabolism, glycolysis), and gene-editing technologies (CRISPR/Cas9, base editing). This review explores the mechanisms of immune dysfunction in *TP53*-mutated MDS and AML while highlighting emerging therapeutic strategies, emphasizing the integration of targeted, metabolic, and immune-modulating therapies as a transformative approach to improve patient outcomes.

## 1 Introduction

The *TP53* gene encodes p53, a tumor suppressor crucial for cell cycle control, apoptosis, genomic integrity, metabolism, immune response, and inflammation. Mutation in *TP53* disrupt these functions, resulting in uncontrolled proliferation, immune evasion, and therapy resistance (1–9). These mutations occur through direct gene mutations or via MDM2 overexpression, a negative regulator of p53 (10). These alterations contribute to genomic instability, impaired apoptosis, and therapy-resistant disease (11–13).

The prevalence of *TP53* mutations varies across cancer types, with an estimated 50% incidence in all cancers (14). While these mutations are prevalent in solid tumors such as ovarian carcinoma (90%), they occur in less than 10% of hematologic malignancies such as AML (15). However, in therapy-related AML, the frequency rises to 50%, highlighting its role in disease progression and treatment resistance (16).

This review investigates the roles that *TP53* mutations play in immunological dysregulation, tumor microenvironment changes, metabolic dysfunction, and treatment resistance. It integrates clinical and preclinical data from immunological, metabolic, and gene-editing methods to highlight potential treatment approaches to *TP53*-mutated MDS and AML.

## 2 Pathogenesis of *TP53* mutations and clonal hematopoiesis in AML and MDS

*TP53* regulates hematopoietic stem cell (HSC) quiescence, proliferation, and the induction of apoptosis, maintaining hematopoietic homeostasis. When *TP53* is mutated, these functions are disrupted, leading to genomic instability, clonal expansion, and the transformation of HSCs into preleukemic stem cells, a key step in the progression to MDS or AML.

### 2.1 Clonal hematopoiesis and TP53 mutations

A major driver of *TP53*-mutated MDS and AML is clonal hematopoiesis, where a single mutated HSC clone expands due to a selective advantage (17). In CHIP, commonly seen in older individuals, *TP53* mutations are among the most significant genetic abnormalities, greatly increasing the risk of progression to hematologic malignancies (18). These mutations promote self-renewal and clonal expansion, driving MDS or AML progression (19).

### 2.2 *TP53* mutation types and their impact on disease progression

The *TP53* mutations occurring through various genetic alterations include the following:

- Deletions: Loss of chromosome 17p, which contains *TP53*, leads to a monoallelic loss, often followed by mutations in the remaining allele, resulting in a complete LOF (20).
- Missense mutations: This is the most frequent type of *TP53* mutation, leading to either dominant-negative effects (where mutant p53 inhibits wild-type function) or GOF mutations that promote oncogenic properties (2, 12–14).
- Truncating mutations: These result in premature stop codons, producing non-functional p53 proteins incapable of exerting tumor-suppressive effects.

Mutation hotspots (codons 175, 245, 248, 249, 273, and 282) correlate with poor AML prognosis (15). *TP53* mutations are more frequent in older patients and those with prior chemotherapy exposure, supporting their role in therapy-related myeloid neoplasms (21).

### 2.3 *TP53* as a distinct molecular entity

The World Health Organization 5th edition and the International Consensus Classification recognize *TP53* mutations as a unique molecular entity (22, 23).

### 2.4 Molecular and clinical determinants of therapy in TP53-mutated MDS/AML

Effective treatment of *TP53*-mutated MDS and AML requires integrating molecular and clinical risk factors. The allelic status of *TP53* mutations has significant prognostic implications. Patients with multi-hit (biallelic) *TP53* mutations exhibit complex karyotyping, primary resistance to chemotherapy, and poor overall survival compared to those with monoallelic mutations (24, 25). Integrating allelic burden into prognostic models such as IPSS-M provides more accurate risk classification and informs intensity of treatment (25, 26). *TP53* mutations co-occur with other genetic mutations, such as RUNX1 or ASXL1, which may further influence disease progression and therapeutic response (24, 27). Updated guidelines, including ELN 2022 and NCCN, now incorporate *TP53* status (including allelic burden) into risk models to enable more personalized, risk-adapted treatment strategies. Patients with biallelic mutations or adverse co-mutations are often directed toward clinical trials or novel agents, while those with monoallelic *TP53* mutations and good fitness may benefit from standard therapies followed by transplant (28–30).

Beyond molecular characteristics, clinical factors including patient age, comorbid conditions, and eligibility for hematopoietic stem cell transplantation remain central to risk assessment and treatment planning. Recent studies have shown that post-transplant survival in *TP53*-mutated MDS/AML is highly dependent on patient fitness and disease status, with poor outcomes in those with high comorbidity, poor performance, or residual disease at transplant (31, 32). These findings highlight the need to align treatment with both clinical fitness and molecular risk: fit patients may pursue intensive approaches like induction and transplant, while unfit patients may be directed to hypomethylating therapy or supportive care.

## 3 Mechanisms of immune evasion in *TP53*-mutated MDS

The role of p53 in tumor immune surveillance has been widely studied across various cancers; p53 enhances anti-tumor immunity by regulating cytokines and tumor recognition (33–36). However, these protective mechanisms are disrupted in *TP53*-mutated MDS and AML, enabling immune evasion and disease progression (4).

*TP53* mutations contribute to genomic instability, leading to a higher mutation burden in tumor cells. Although TP53-mutant cancers generate neoantigens, TP53-mutated MDS/AML typically have lower tumor mutational burdens, suggesting immune evasion mechanisms hinder anti-tumor responses (37).

**Figure 1:**
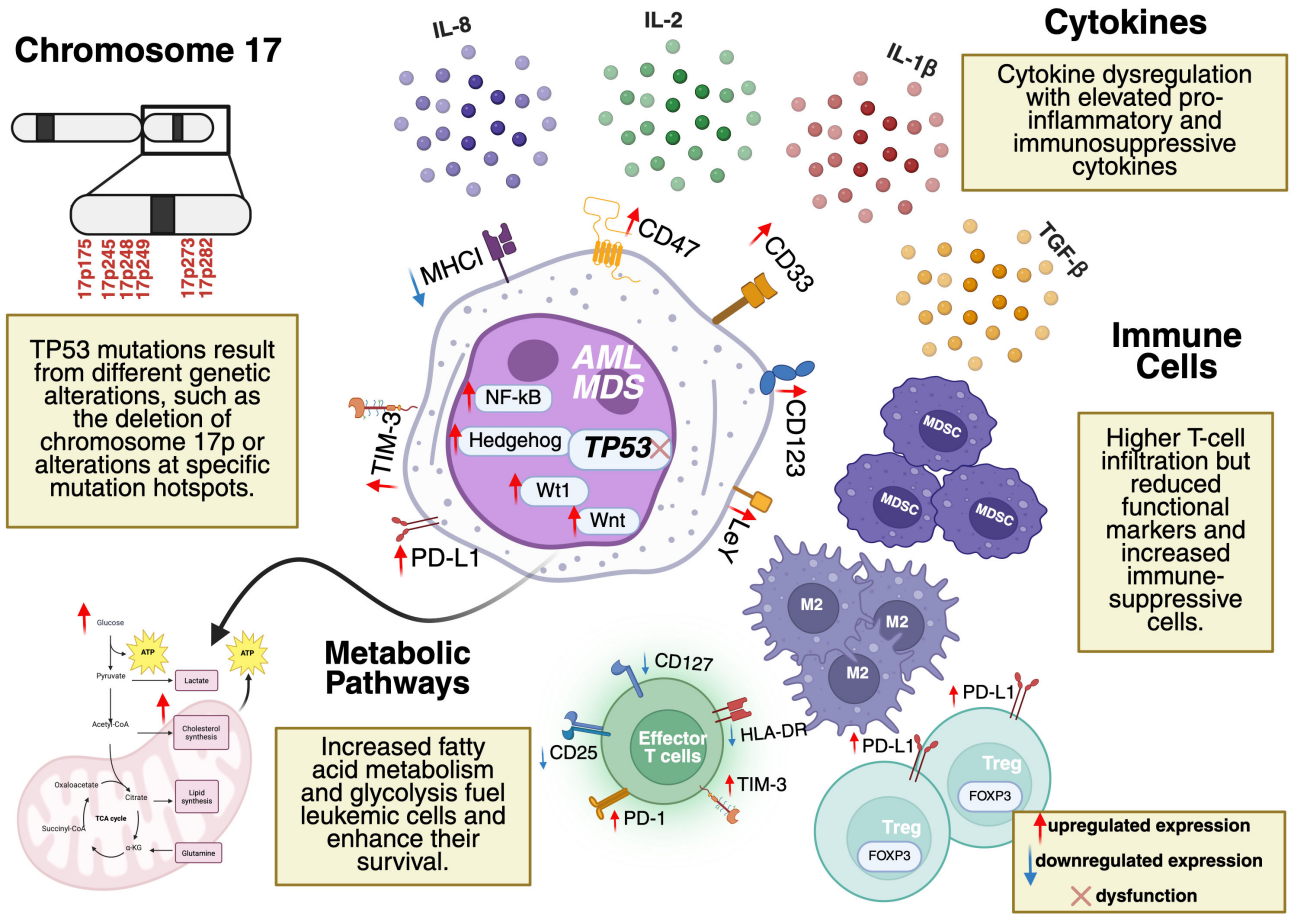
Tumor microenvironment (TME) in TP53-mutated AML and MDS. Key features include cytokine dysregulation (elevated IL-1β, IL-8, TGF-β), immune suppression (Tregs, MDSCs, reduced effector T-cell functionality), and metabolic reprogramming (enhanced glycolysis, fatty acid metabolism). Mutation hotspots in TP53 (codons 175, 245, 248, 249, 273, 282) are located on Chromosome 17p. Dysregulated pathways (e.g., NF-κB, Hedgehog, Wnt) and overexpressed markers (e.g., CD123, CD33, CD47) drive leukemic progression and represent potential therapeutic targets. Figure created by the author using BioRender.

A key immune escape mechanism is impaired antigen presentation due to the downregulation of major histocompatibility complex class I (MHC I) molecules essential for presenting tumor antigens to cytotoxic T lymphocytes (CTLs) (38). The reduced expression of MHC I decreases the ability of the immune system to recognize and eliminate malignant cells, contributing to an immunosuppressive tumor microenvironment (TME) that facilitates leukemic progression. Additionally, *TP53*-mutated MDS and AML are characterized by cytokine dysregulation, immune suppression through regulatory T cells (Tregs) and myeloid-derived suppressor cells (MDSCs), and metabolic reprogramming, including increased glycolysis and fatty acid metabolism. Beyond immune suppression, metabolic adaptations further reinforce the leukemic microenvironment, creating additional barriers to effective immune responses (**Figure 1**).

### 3.1 Cytokine dysregulation and oncogenic signaling

In *TP53*-mutated MDS and AML, cytokine dysregulation is key in shaping the immunosuppressive TME. Elevated levels of pro-inflammatory and immunosuppressive cytokines such as IL-1β, IL-2, IL-8, and TGF-β contribute to leukemic cell survival and immune dysfunction (39). These cytokines interact with oncogenic signaling pathways, including Hedgehog, Wnt, and NF-κB, which further promote immune evasion.

#### 3.1.1 Hedgehog signaling in immune evasion

The Hedgehog (Hh) pathway is critical for HSC regulation and differentiation but becomes dysregulated in *TP53*-mutated AML and MDS, leading to self-renewal of malignant stem cells and suppression of anti-tumor immunity (40)​. Aberrant Hh signaling promotes

- Accumulation of immunosuppressive M2 macrophages, which suppress anti-tumor immunity.
- PD-L1 overexpression, leading to T-cell exhaustion and immune evasion.
- Inhibition of effector T-cell function, further reducing immune-mediated tumor elimination (41, 42).

#### 3.1.2 Wnt signaling and immunosuppressive microenvironment

Wnt signaling is fundamental to stem cell proliferation and differentiation but is often hyperactivated in *TP53*-mutated malignancies, promoting leukemia stem cell expansion and therapy resistance (43). Additionally, Wnt signaling contributes to immune evasion by

- Inducing the accumulation of Tregs and tumor-associated macrophages, which suppress anti-tumor immunity (44, 45).
- Driving T-cell exhaustion, leading to reduced CTL activity against leukemic cells (44, 45).

Currently, no clinical trials have specifically targeted Wnt inhibitors in hematologic malignancies, highlighting a gap in therapeutic development.

#### 3.1.3 NF-κB signaling and pro-tumor immune regulation

Aberrant activation of NF-κB signaling in *TP53*-mutated AML and MDS contributes to immune evasion through

- Upregulation of anti-apoptotic proteins (e.g., BCL-XL, MCL-1) that sustain malignant clones.
- Increased production of IL-6 and TNF-α, which promote leukemia progression and chemotherapy resistance.
- Expansion of immunosuppressive cell populations, including Tregs and MDSCs, which inhibit CTLs and natural killer (NK) cells (46)​.

Due to the role of NF-κB in leukemic survival and immune suppression, its inhibitors combined with immune-modulating agents may benefit TP53-mutated AML/MDS.

### 3.2 Immune checkpoints and T-cell dysfunction

*TP53*-mutated AML and MDS are associated with dysfunctional T-cell immunity despite increased immune cell infiltration in the bone marrow. Studies have shown that *TP53*-mutated AML patients exhibit

- Reduced functional memory T-cell markers (e.g., CD127, CD25, HLA-DR) (39).
- Increased regulatory T-cell (Treg) populations, which suppress anti-tumor immunity (47, 48).
- Upregulated metabolic pathways (e.g., glycolysis, fatty acid metabolism, and oxidative phosphorylation) that enhance Treg-mediated immune suppression (49).

Additionally, CD8+ CTLs in *TP53*-mutated AML show hallmarks of exhaustion, including increased expression of mucin-domain containing-3 (TIM3) and PD-1 (47). The upregulation of immune senescence markers, such as PD-L1 and T-cell immunoreceptors with Ig and ITIM domains (TIGIT), further promotes immune evasion by preventing effective T-cell activation (39)

A bone marrow biopsy study evaluating T-cell infiltration in relapsed *TP53*-mutated AML found that PD-L1 expression was significantly higher in CD8+ T cells from patients with multiple relapses compared to newly diagnosed cases (47). This suggests that immune checkpoint dysregulation plays a progressive role in disease evolution and resistance to therapy.

## 4 Therapeutic approaches

For over 3 decades, chemotherapy has remained the standard treatment for AML and MDS. However, patients with *TP53*-mutated AML and MDS exhibit significantly lower response rates to conventional therapies, including cytotoxic chemotherapy, hypomethylating agents (HMAs) such as azacitidine and decitabine, and venetoclax-based regimens. Compared to patients with wild-type *TP53*, those harboring *TP53* mutations have a markedly worse prognosis, with a median overall survival (OS) of only 5–10 months (50).

Currently, allogeneic HSCT (allo-HSCT) remains the only potentially curative option for *TP53*-mutated MDS and AML patients eligible for transplantation. However, outcomes remain suboptimal, as *TP53* mutations are associated with higher relapse rates and poor long-term survival following transplantation (51). HMAs are frequently used as cytoreductive therapy before allo-HSCT and as monotherapy in patients ineligible for transplant due to advanced age or comorbidities (52).

Despite these treatment strategies, effective therapies for *TP53*-mutated disease remain an urgent unmet need. The development of novel targeted therapies and immunotherapies has the potential to overcome treatment resistance and improve survival outcomes. Emerging approaches, including agents that restore TP53 function, immune-based therapies, and metabolic pathway inhibitors, represent promising avenues for improving disease management and enhancing survival and quality of life in these high-risk patients.

**Table 1** summarizes current and investigational therapeutic strategies, including their mechanisms of action and clinical progress.

**Table 1:** Summary of therapeutic strategies for *TP53*-Mutated MDS and AML.

Therapy	Mechanism of action	Current phase	Clinical outcomes	Challenges/limitations	Citation
RG7112	Restores p53 function by inhibiting MDM2 degradation	Phase I	Increased p53 expression; activation of downstream genes.	Resistance mechanisms; limited efficacy in *TP53*-mutated hematologic malignancies.	(53)
APR246	Reactivates mutant *TP53*, restoring wild-type function	Phase III	Improved overall response rate when combined with azacitidine in *TP53*-mutated patients.	Variable patient responses; resistance mechanisms; uncertain durability of response.	(54)
Hedgehog pathway (e.g., Glasdegib)	Inhibits Hedgehog signaling, reducing leukemia stem cell survival	Phase III	FDA-approved for AML in combination with chemotherapy; mixed results in Phase III trials.	Toxicity concerns; no significant improvement in overall survival in late-stage trials.	(55, 56)
Wnt Pathway Inhibitors	Blocks Wnt signaling, reducing leukemic stem cell self-renewal	Preclinical	Potential to inhibit leukemia progression	No clinical trials yet in hematologic malignancies	(57)
NF-κB Inhibitors	Suppresses NF-κB-driven tumor survival and immune evasion	Preclinical	Reduces pro-inflammatory cytokine production	No approved agents, toxicity concerns	(46)​
Anti-PD-1 + Azacitidine	Immune checkpoint blockade, enhances T-cell response	Phase II	33% overall response rate with 6.3 OS months	Increased immune-related toxicities; modest survival benefit.	(58)
Anti-CD47 (Magrolimab)	Blocks “don’t eat me” signal, enhances macrophage-mediated clearance	Phase III	Phase Ib trials showed promising results; Phase III trial halted due to futility.	Trial Terminated due to futility	(59, 60)
Magrolimab + Venetoclax + Azacitidine		Phase II Phase III undergoing	41% CR rate in *TP53*-mutated patients; OS of 16.3 months in responders.	Toxicity concerns; requires further validation in larger trials.	(61, 62)
VIP-Receptor Antagonists	Blocks VIP-enhanced cell immunity in AML	Preclinical	Increased leukemia clearance in murine models	No human clinical trials yet	(63)
Anti-CD3 + CD123 (Flotetuzumab)	Targets CD123 on leukemic cells, enhances T-cell cytotoxicity	Phase I/II	Improved immune infiltration; limited OS benefit in *TP53*-mutated patients.	Limited survival benefit; optimization needed for broader use.	(39, 61)
Anti-CD123-CAR T-Cell Therapy		Phase I/II	Reduction in leukemic burden in pediatric and adult AML; well-tolerated in early trials.	T-cell exhaustion and antigen escape reduce long-term efficacy.	(64, 65)
Anti-CD123 CAR T-cells + Azacitidine		Preclinical	Increased CD123 expression and improved CAR T-cell function.	Translation to clinical trials required; potential toxicity in combination	(66, 67)
CD33-CAR T-Cell Therapy	Targets CD33 on myeloid leukemia cells	Phase I/II	Early efficacy reported; some cases achieved MRD-negative status.	Antigen escape; off-target toxicity; limited durability.	(68)
Emerging CAR T-Targets (WT1, Tim-3, and LeY)	Targets leukemia-associated antigens	Preclinical	Selective targeting of leukemia cells in preclinical studies.	Development and validation in clinical settings needed.	(69–71)
HSPC Epitope Mapping	Identifies unique leukemia cell markers for CAR-T precision	Preclinical	Potential to minimize off-target toxicity	No clinical data available	(72)
Fatty Acid Metabolism Inhibitors	Disrupts lipid metabolism to suppress leukemia cell survival	Preclinical	Associated with improved immune infiltration	Lacks clinical validation	(73)
Glycolysis Inhibitors	Blocks glycolytic pathways to limit leukemia proliferation	Preclinical	Restores T-cell function, reduces tumor growth	Safety and efficacy in AML remain unproven	(74)
CRISPR/Cas9 Gene Editing	Corrects *TP53* mutations at the genomic level	Preclinical	Restores wild-type p53 function *in vitro*	Delivery challenges, potential off-target effects	(75–77)
Base Editing/Prime Editing	Precise correction of *TP53* mutations without double-strand breaks	Preclinical	More targeted than CRISPR/Cas9	Requires clinical validation	(78)

### 4.1 Targeting TP53 mutations directly

Directly targeting *TP53* mutations represents a promising therapeutic strategy, aiming to restore or enhance p53 function in *TP53*-mutated AML and MDS. Several small-molecule agents are currently in clinical trials, focusing on either stabilizing p53 expression or reactivating mutant p53 to restore its tumor-suppressive functions.

MDM2 inhibitors, such as RG7112 and idasanutlin, are designed to prevent p53 degradation by inhibiting MDM2, a negative regulator of p53. Blocking MDM2-p53 interactions enables p53 accumulation and apoptotic pathway activation. MDM2 inhibitors have shown preclinical efficacy. However, early-phase clinical trials in leukemia patients have reported limited success, as their effectiveness depends on the presence of wild-type *TP53*, which is frequently absent in *TP53*-mutated AML and MDS (53).

APR246 (eprenetapopt) reactivates mutant p53, restoring apoptosis and cell cycle arrest. In multiple clinical trials, APR246 combined with azacitidine has demonstrated enhanced anti-tumor activity, leading to higher response rates in *TP53*-mutated AML and MDS compared to azacitidine alone (79–81). APR246 is now in Phase III trials evaluating response rates and durability (54).

Given the complexity of *TP53* mutations and resistance mechanisms, combining *TP53*-reactivating agents with immunotherapies or other targeted treatments is being actively investigated. These combinations may enhance disease control and survival in patients with TP53 mutations (82).

### 4.2 Pathway-targeted therapies

Targeting dysregulated signaling pathways represents a promising therapeutic approach in *TP53*-mutated AML and MDS, particularly given their role in leukemia stem cell survival, immune evasion, and therapy resistance. Several pathways, including Hh, Wnt, and NF-κB, have been implicated in disease progression, and efforts to develop targeted inhibitors are ongoing.

#### 4.2.1 Hedgehog pathway inhibition

The Hh signaling pathway, involving key components such as SMO, SHH, and GLI3, is critical in stem cell maintenance and differentiation. Aberrant activation of this pathway has been associated with resistance to HMAs in AML and MDS, promoting leukemia stem cell survival and self-renewal (83).

Preclinical studies have shown that combining Hh inhibitors with 5-azacytidine exhibit synergistic cytotoxic effects in AML models (84, 85). Among these inhibitors, glasdegib, an SMO inhibitor, has shown promising activity in preclinical models and early-phase clinical trials, including in patients with TP53-mutated AML (86–88). In the Phase II BRIGHT AML 1003 trial, glasdegib combined with low-dose cytarabine (LDAC) improved complete remission (CR) rates, leading to FDA approval (87).

However, the Phase III BRIGHT AML 1019 trial failed to demonstrate an improvement in OS, limiting glasdegib’s clinical impact in a broader population (55, 56). Differences in trial design, treatment intensity, or patient stratification including, co-mutation profiles, karyotype complexity, TP53 variant allele frequency (VAF), and functional status, may have influenced therapeutic outcomes. The Phase III enrolled a larger population and combined glasdegib with intensive chemotherapy compared to the smaller Phase II that used low-dose chemotherapy. These findings underscore the need for future studies to incorporate comprehensive molecular and clinical stratification to evaluate glasdegib and other Hh inhibitors in combination with targeted therapies for TP53-mutated AML and MDS.

### 4.3 Immune checkpoint inhibitors and immunotherapy

The use of immune checkpoint inhibitors targeting PD-1 (programmed cell death protein 1) and PD-L1 (programmed cell death ligand 1) has revolutionized cancer treatment, particularly in solid tumors. However, their efficacy in AML and MDS, particularly in *TP53*-mutated cases, has been limited. While PD-1/PD-L1 inhibitors combined with HMAs such as azacitidine have shown enhanced immune responses, overall clinical outcomes remain modest (89, 90).

#### 4.3.1 PD-1/PD-L1 inhibitors in TP53-mutated AML and MDS

A Phase II trial evaluating nivolumab (PD-1 inhibitor) combined with azacitidine in relapsed/refractory AML reported a 33% overall response rate and a median OS of 6.3 months but with limited long-term benefit (58). Similarly, in higher-risk MDS, combination therapies involving PD-1/PD-L1 inhibitors with HMAs have not demonstrated significantly improved survival compared to HMAs alone (91). Additionally, these therapies have been associated with increased immune-related adverse events, including cytopenias and infections, particularly in older and frail patients, further complicating their clinical application.

The limited efficacy of immune checkpoint blockade in *TP53*-mutated AML and MDS may be attributed to several factors, including

- Intrinsic resistance mechanisms such as mutations in PD-1/PD-L1 signaling pathways.
- Defective antigen presentation due to MHC dysfunction, reducing T-cell activation.
- An immunosuppressive bone marrow TME suppressing T-cell infiltration and function (92).

Given these challenges, combination strategies are being explored to overcome immune resistance. For instance, integrating PD-1 inhibitors with chimeric antigen receptor T-cell therapy has shown promise in other hematologic malignancies, such as diffuse large B-cell lymphoma with *TP53* alterations (93).

#### 4.3.2 Targeting CD47: “Don’t Eat Me” signal blockade

Beyond PD-1/PD-L1 inhibitors, other immune-modulating agents are being investigated. One such approach targets CD47, a key regulator of the “don’t eat me” immune evasion mechanism.

Magrolimab, a CD47-blocking antibody, enhances macrophage-mediated phagocytosis, promoting immune-mediated leukemia clearance. In a Phase Ib trial in MDS patients, magrolimab combined with azacitidine achieved a 40% CR rate and a median OS of 16.3 months, with a 2-year OS of 77% in patients undergoing allogeneic stem cell transplantation (59). However, despite these promising early-phase results, a Phase III trial evaluating magrolimab with azacitidine was discontinued due to futility (60).

Conversely, a Phase II study examining a triple regimen of venetoclax, azacitidine, and magrolimab in newly diagnosed and relapsed/refractory AML demonstrated encouraging response rates with manageable toxicity. Among patients with *TP53* mutations, the CR rate was 41%, with a 1-year OS of 53%, compared to 83% in patients with wild-type *TP53*. A Phase III trial is currently underway to further evaluate this triplet combination in newly diagnosed AML (61, 62).

#### 4.3.3 Emerging checkpoint targets: vasoactive intestinal polypeptide signaling

In 2024, a study published in Blood identified VIP signaling as a potential immune checkpoint in *TP53*-mutated AML. VIP was found to be overexpressed in CD34-high and *TP53*-mutated AML cells, contributing to immune suppression by interacting with VPAC1 receptors on myeloid cells and VPAC2 receptors on lymphoid cells (94).

Interestingly, in non-mutated AML, VIP signaling was correlated with enhanced opsonization, suggesting that VIP may influence the efficacy of antibody-based therapies (94). Preclinical models of murine AML treated with a VIP receptor antagonist showed immune-cell-mediated leukemia eradication, with long-term survival rates of 40% in VIP-negative cases and 75% in VIP-positive models (63).

These findings highlight VIP signaling as a novel immunotherapeutic target in *TP53*-mutated AML, warranting further exploration in clinical trials.

### 4.4 CAR T-cell and NK-cell therapies

Adoptive cell therapies, including chimeric antigen receptor (CAR) T-cell therapy and NK-cell therapy, have emerged as promising therapeutic strategies in *TP53*-mutated AML and MDS. These therapies harness the immune system to selectively target tumor-associated antigens overexpressed in leukemic cells, enabling immune-mediated elimination. Among the most well-characterized targets in AML and MDS are CD123 and CD33. Both are highly expressed in leukemic stem cells and blasts, making them ideal candidates for CAR-based therapies.

Despite promising preclinical data, the clinical translation of CAR T-cell therapies in AML and MDS remains challenging due to factors such as antigen heterogeneity, TME-induced immunosuppression, and therapy resistance (95). Strategies to improve efficacy, including dual-targeting approaches, CAR modifications, and combination therapies, are currently being explored.

#### 4.4.1 CD123-CAR T-cell therapy

CD123, the alpha chain of the interleukin-3 receptor, is widely overexpressed in AML and MDS, particularly on leukemia stem cells, making it a key target for immunotherapies (96). Several therapeutic approaches have been developed to exploit CD123, including bispecific antibodies and CAR T-cell therapies.

##### 4.4.1.1 Bispecific CD123/CD3 therapy

Flotetuzumab, a dual-affinity retargeting antibody, simultaneously binds CD3 on T cells and CD123 on leukemia cells, enabling T-cell-mediated leukemia cell destruction. A clinical study evaluating flotetuzumab in 35 patients with relapsed/refractory AML, including 14 with *TP53* mutations, demonstrated increased immune infiltration in patients with *TP53* mutations. However, these patients had a median OS of only 4.5 months, compared to 18.5 months in *TP53*-wild-type cases, underscoring the persistent poor prognosis associated with *TP53* mutations despite immunotherapy (39, 61).

##### 4.4.1.2 CD123-CAR T-cell therapy

CD123-CAR T-cell therapy employs genetically modified T cells engineered to recognize and eliminate CD123-expressing leukemia cells. In a Phase I trial of pediatric relapsed/refractory AML, CD123-CAR T-cell therapy demonstrated significant anti-tumor activity with a favorable safety profile, as no Grade 2 or higher cytokine release syndrome (CRS) or neurotoxicity was observed (64). In adult patients, the therapy led to a reduction in tumor burden at dose level 2, but sustained responses were limited due to poor CAR T-cell persistence (65). Consequently, ongoing clinical trials are incorporating FCA lymphodepletion regimens and prophylactic tocilizumab to mitigate CRS and improve CAR T-cell durability.

##### 4.4.1.3 Combination strategies to enhance CD123-CAR T-cell efficacy

Combining CD123-CAR T-cell therapy with HMAs, such as azacitidine, is being explored to further enhance efficacy (66, 67); this approach is hypothesized to increase CD123 expression in leukemia cells, improving CAR T-cell recognition and cytotoxicity while also enhancing T-cell activation to strengthen anti-leukemic effects. These findings support the continued development of combination strategies integrating CAR T-cell therapy with epigenetic modulators to improve outcomes in *TP53*-mutated AML (59).

#### 4.4.2 CD33-CAR T-cell therapy

CD33 is another widely expressed myeloid antigen in AML and MDS, making it a valuable target for CAR T-cell therapy. In a Phase I/II clinical trial, CD33-CAR T-cell therapy demonstrated early efficacy, with some patients achieving CR with minimal residual disease negativity (68).

However, significant challenges remain, including manufacturing delays associated with autologous CAR T-cell production and rapid disease progression, which can outpace CAR T-cell expansion and activity (97). To address these limitations, ongoing research is focusing on optimizing CAR constructs to enhance persistence and reduce T-cell exhaustion while also exploring allogeneic “off-the-shelf” CAR T-cell therapies to mitigate manufacturing delays and improve accessibility. Despite these obstacles, CD33-targeted CAR T-cell therapy remains an area of active investigation, with efforts directed toward refining its clinical applicability in high-risk *TP53*-mutated AML and MDS.

#### 4.4.3 Emerging targets and challenges

Beyond CD123 and CD33, additional tumor-associated antigens are being explored for CAR T-cell therapy in *TP53*-mutated AML and MDS. Notable targets include WT1, a transcription factor overexpressed in leukemia stem cells; Tim-3, a checkpoint receptor involved in immune evasion; and LeY, an antigen found in a subset of myeloid leukemias. These are thus promising candidates for immunotherapy (69–71).

Despite their potential, translating CAR T-cell therapy into clinical use faces challenges such as antigen escape, off-target toxicity, and T-cell exhaustion. Leukemia cells can downregulate target antigen expression, leading to relapse, while shared antigen expression with normal hematopoietic cells increases the risk of prolonged myelosuppression. Additionally, T-cell exhaustion due to prolonged activation remains a barrier to achieving durable responses (98).

To overcome these limitations, researchers are exploring multiplex CAR T-cell designs to target multiple antigens simultaneously, reducing antigen escape. Hematopoietic stem and progenitor cell (HSPC) epitope mapping is being investigated to help differentiate leukemia cells from healthy cells, minimizing off-target toxicity (72). Additionally, combining CAR T-cell therapy with immune checkpoint blockades, such as PD-1 inhibitors, may enhance T-cell function and persistence, potentially leading to more sustained anti-leukemic responses.

### 4.5 Modulating the TME

The TME is crucial in the progression and treatment resistance of *TP53*-mutated AML and MDS. In these malignancies, the TME is highly immunosuppressive, driven by pro-tumorigenic cytokines, metabolic reprogramming, and immune evasion mechanisms. A key factor is IL-8, which promotes leukemic cell proliferation and therapy resistance (99, 100). Preclinical studies suggest that neutralizing IL-8 with specific antibodies can reduce AML cell growth and enhance sensitivity to chemotherapy, highlighting its potential as a therapeutic target (100).

Beyond cytokine dysregulation, metabolic alterations within the TME further contribute to immune evasion and disease persistence. Dysregulated lipid metabolism and glycolysis provide crucial energy sources for leukemic cells while supporting the survival of immunosuppressive populations. Strategies that disrupt these metabolic dependencies may help reshape the TME, enhancing anti-tumor immune responses and improving treatment outcomes for *TP53*-mutated AML and MDS.

#### 4.5.1 Lipid metabolism

Lipid metabolism alterations are increasingly recognized as key contributors to leukemic progression and therapy resistance in *TP53*-mutated AML. A 2024 multi-omic analysis published in Blood found that specific *TP53* hotspot mutations (R175H, R273H) were associated with enhanced oxidative phosphorylation and a higher prevalence of primitive leukemia stem and progenitor states (101). Conversely, *TP53*-wild-type and R248Q-mutated AML cells displayed enrichment in cellular senescence, heme metabolism, and fatty acid metabolism pathways, suggesting that *TP53* mutations may drive metabolic reprogramming, promoting stemness and aggressive disease behavior (101).

In addition to its role in leukemia cell survival, lipid metabolism influences immune cell function within the TME. A 2022 study in Lipid Insights identified a fatty acid metabolism-related signature correlating with immune cell infiltration and clinical outcomes in AML, indicating its prognostic value (102). This suggests that targeting fatty acid metabolism could not only disrupt leukemic cell survival but also enhance anti-tumor immunity.

The cholesterol pathway has also been implicated in AML resistance mechanisms. A recent Blood study found that *TP53*-mutated AML cells exhibited increased cholesterol metabolism, contributing to immune evasion and reduced CAR T-cell efficacy (103). Notably, simvastatin-mediated cholesterol pathway inhibition improved CAR T-cell function, reducing T-cell exhaustion and enhancing tumor clearance (73). These findings suggest that combining CAR T-cell therapy with lipid metabolism inhibitors may improve responses in *TP53*-mutated AML and MDS, warranting further clinical investigation.

#### 4.5.2 Glycolysis

Glycolytic reprogramming is another hallmark of *TP53*-mutated AML, enabling leukemic cells to sustain rapid proliferation and evade immune surveillance. A 2024 study published in Frontiers in Pharmacology examined the metabolic and physiological hallmarks of *TP53*-mutated AML, highlighting an upregulated glycolysis pathway as a key driver of chemoresistance and immune evasion (104, 105).

The reliance on glycolysis in *TP53*-mutated AML suggests that targeting this metabolic pathway may enhance therapeutic efficacy. Preclinical models indicate that inhibiting glycolysis can reduce leukemia cell proliferation, sensitize AML cells to standard chemotherapy, and restore T-cell function within the TME (74). However, clinical validation is still required to determine the safety and efficacy of glycolysis inhibitors in *TP53*-mutated AML and MDS.

### 4.6 Gene-editing technologies

Advances in gene-editing technologies, particularly CRISPR/Cas9, represent a promising frontier in the treatment of *TP53*-mutated MDS and AML. These tools aim to correct *TP53* mutations at the genomic level, potentially restoring normal p53 function and immune regulation. Preclinical studies have demonstrated success in repairing *TP53* mutations, reinstating wild-type p53 activity, and selectively eliminating p53-deficient cells while sparing normal ones (75–77). Additionally, computational tools such as the Computational CRISPR Strategy improve precision, while emerging techniques such as base and prime editing enhance mutation correction (78).

Despite these advances, several challenges remain, particularly in safe and efficient delivery to HSPCs. Current methods, including viral vectors and lipid nanoparticles, pose risks of toxicity, immunogenicity, and off-target effects, potentially leading to genomic instability or the emergence of new oncogenic drivers (69). Timely and scalable gene-editing methods are necessary given the rapid progression of *TP53*-mutated AML.

Ongoing research is focused on improving guide RNA design, developing high-fidelity Cas9 variants, and refining targeting specificity to minimize off-target effects. While gene editing holds long-term therapeutic potential, its clinical success will depend on advances in delivery methods, safety validation, and successful clinical translation.

## 5 Conclusion

Treating *TP53*-mutated MDS and AML remains challenging due to poor prognoses, resistance to standard therapies, and limited treatment options. However, recent advances in understanding the role of *TP53* mutations in immune dysregulation and tumor progression have opened new avenues for therapeutic innovation. These approaches, including targeted therapies, immunotherapies, metabolic interventions, and gene editing, are emerging as promising alternatives. Enhancing combination therapies, improving immune modulating, and advancing gene-editing delivery will be essential for improving patient outcomes.
